# Perceived Health in Patients with Primary Immune Deficiency

**DOI:** 10.1007/s10875-015-0196-7

**Published:** 2015-10-09

**Authors:** Filiz Odabasi Seeborg, Roann Seay, Marcia Boyle, John Boyle, Christopher Scalchunes, Jordan Scott Orange

**Affiliations:** Department of Pediatrics, Section of Immunology, Allergy and Rheumatology, Baylor College of Medicine and Texas Children’s Hospital, 1102 Bates Ave. Suite 330, Houston, TX 77030 USA; The University of Texas Health Science Center, School of Public Health, Houston, TX USA; Immune Deficiency Foundation, Towson, MD USA

**Keywords:** Perceived health, health status, primary immune deficiency, health survey, immunoglobulin

## Abstract

**Purpose:**

Perceived health (PH) is a subjective measure of global health of individuals. While many studies have evaluated outcomes in patients with primary immune deficiency (PID), published literature evaluating PH among patients with PID is sparse. We evaluated the results of the largest self-reported survey of patients with PID to determine the factors that may contribute to differences in PH.

**Methods:**

Data from a National Survey of Patients with Primary Immune Deficiency Diseases conducted by the Immune Deficiency Foundation was studied. Multivariate logistic regression was employed for data analysis.

**Results:**

Thirty percent of the patients perceived their health status as excellent or very good (EVG), 31 % as good (G), and 39 % as fair, poor or very poor (P). Older patients were less likely to have EVG-PH compared to G-PH. Ones with college degrees were more likely to have P-PH compared to G-PH, and less likely to have EVG-PH. Patients who were acutely ill and hospitalized in the past 12 months, ones with limited activity, and chronic diseases, were more likely to have P-PH compared to G-PH. Patients with “on demand” access to specialty care and ones on regular IVIG had higher OR of having EVG-PH as opposed to G-PH. Patients cared for mostly by an immunologist were less likely to have P-PH compared to G-PH.

**Conclusions:**

Our results emphasize the importance of PH in clinical practice. We suggest that recognizing the factors that drive PH in patients with PID is important for the development of disease prevention and health promotion programs, and delivery of appropriate health and social services to individuals with PID.

## Introduction

Perceived health is a subjective measure of global health of individuals. There is a clear difference between the real PH assessments and questions regarding the prevalence of diseases. The PH assessment reflects individuals’ integrated PH, including the biological, psychological, cultural, and social dimensions, which is inaccessible to any external observer [1–3]. In other words, they cannot be easily detected by health care professionals. Perceived health status has been extensively used in epidemiologic studies as a broad indicator of health-related well-being [4–6]. It is an important predictor of a number of outcomes, such as new morbidity, functional ability, health care utilization [7,8], recovery from illness [9], and physician ratings of health [10]. It may encompass aspects that are difficult to capture clinically, such as incipient disease, physiological and psychological reserves, and social function [11–13]. Moreover, it is an important and reliable indicator of quality of care as well as patient satisfaction, and quality improvement [14]. Existing evidence supports that patients in better health tend to report greater satisfaction with their health status and health care than patients in poor health [15]. Numerous epidemiological studies have reported an association between PH and mortality from all causes [3,16–19] while others have shown it to be associated with specific chronic diseases such as musculoskeletal, cardiovascular, and psychiatric disorders [20]. Perceived health may be influenced by age and gender differences as well as social, environmental and personal factors [1,21]. For example, individuals who consider themselves to be in poor health may be more likely depressed, may have disabilities, may be leading less productive and fulfilling lives, or may not be receiving the health care that they need.

Perceived health, also known as “self-assessed health”, “self-rated health”, “self-evaluated health”, or “subjective health”, has been evaluated and described in several patient populations with chronic conditions including systemic lupus erythematous [22], chronic obstructive pulmonary disease [23], inflammatory bowel disease [24], asthma [25], and cancer [14]. However, published literature evaluating patient-reported outcome measures related to patients with PID is sparse [26,27], and none have specifically analyzed PH. As a chronic disease, complications of PID bring about major challenges to health and social life of individuals affected by it. Understanding the health status of this population and the factors driving it is important for the development of disease prevention and health promotion programs, as well as the delivery of appropriate health and social services to individuals with PID. Therefore, we aimed to describe general characteristics and health of a national U.S. sample of patients with a variety of PID diagnoses, and define the variables that influenced PH among patients with PID.

## Methods

### Data Source

Investigations were based on data obtained from the Second National Survey of Patients with Primary Immune Deficiency Diseases in America, which was conducted by the Immune Deficiency Foundation (IDF) in 2002. The IDF contact database provided the first stage in the construction of the sampling frame. This large national sample consisted of 5922 adults and children with PID across U.S. Patients were mailed a two-page self-administered questionnaire, along with a cover letter explaining the purposes of the survey. A total of 1587 individuals completed and returned the questionnaire (26.8 %). 49 cases were identified as deceased patients with PID, and the remainder was patients without PID but with other diagnoses such as autoimmune disease. Therefore data were collected from only 1526 patients with PID.

### Variables of Interest

The outcome measure, perceived health status, was assessed on a 6-point Likert scale (1 = excellent, 2 = very good, 3 = good, 4 = fair, 5 = poor, 6 = very poor) based on the question “Would you describe his/her current health status as excellent, very good, good, fair, poor, or very poor?”. The excellent and very good response categories, as well as the fair to very poor categories were combined, creating three comparison groups: excellent to very good (EVG), good (G), and fair to very poor which is referred to hereafter as poor-rated (P) health. Each indicator of PH status was coded according to a trinomial outcome variable (0 = good, 1 = excellent /very good, 2 = fair/poor/very poor). Independent variables were grouped into the following: Sociodemographic, PID diagnosis (type), current health status, comorbidities, health care access, and IVIG treatment. The sociodemographic variables included: age, gender, education status (less than college, college and higher), questionnaire respondent (adult patient, parent/caregiver, both), employment status (unemployed, employed, other), and number of children with PID in the household (none vs. at least one). The very first question asked who the respondent was in relation to a patient with a PID. If there were no children in the household with PID, it was instructed that patients with PID should answer about themselves. If they were not patients with PID then it was instructed that they should answer questions about the oldest child in the household with PID.

**Fig. 1:**
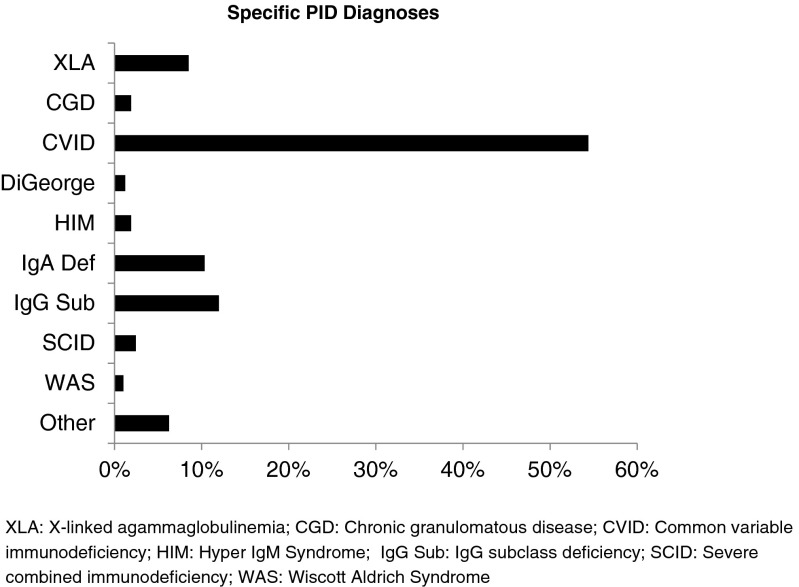
National Survey of Patients with Primary Immune Deficiency, 2002: Primary Immune Deficiency Diagnoses

Patient sample had a number of PID diagnoses, and included common variable immunodeficiency (CVID), IgA deficiency, IgG subclass deficiency, X-linked agammaglobulinemia (XLA), severe combined immunodeficiency (SCID), chronic granulomatous disease (CGD), Hyper IgM Syndrome (HIM), Wiskott Aldrich Syndrome (WAS), and DiGeorge Syndrome (DGS) (Fig. 1). Common variable immunodeficiency accounted for the majority of the patient sample (about 54 %), followed by IgG subclass and IgA deficiencies, and all other diagnoses were smaller proportions. Because of the large distribution differences within PID diagnoses, and CVID comprising the majority of the diagnoses we observed a better fit in logistic regression modeling when diagnoses were reclassified in two groups (CVID vs. non-CVID). Therefore items related to PID diagnosis were assessed as follows: having CVID (yes/ no), having a family member with PID (yes/ no), having infections prior to diagnosis with PID (yes/ no), reason for initial testing for PID (family history, routine check-up, recurrent infections, unusual infections, serious infections), and number of hospitalizations prior to diagnosis with PID (none vs. at least one). Current health was elucidated through three questions and included: having acute illness in the past 12 months (yes/ no), hospitalization in the past 12 months (yes/ no), and limitation of physical activity (none, slight, moderate, severe). Comorbidities included: having permanent functional impairment (yes/ no), other chronic disease (yes/ no), cancer/leukemia (yes/ no), hepatitis (yes/ no), or neurological disease (yes/ no). Health care access was assessed by five questions and included: physician visited most often for health care (specialist in immunology vs. other specialist), setting of primary care visit (private office vs. others), having “on demand” access to specialist care as needed (yes/ no), having visited immunologist in the past 12 months (yes/ no), insurance status (private vs non-private). Treatment status with IVIG was assessed by the following questions: ever receiving IVIG on a regular basis (yes/no), length of regular IVIG treatment, current treatment status with IVIG (yes/no), frequency of IVIG treatment (every 2 weeks or more often, every 3 weeks, every 4 weeks, every 5 weeks, every 6 weeks or more), side effects of IVIG treatment (yes/no). Because the data related to IVIG treatment was sparse (<15 %) (except for the question that addressed whether the patient has ever received regular treatment with IVIG), other variables of IVIG treatment were excluded from the analysis.

### Statistical Analysis

To model our multinomial scaled outcome, we employed the backwards elimination method to build our multinomial logistic regression model using Stata 12 (StataCorp, Inc., College Station, TX). We first conducted a univariable analysis of all the non-sparse variables, defined as variables with less than an 85 % response rate, and retained the covariates with a *p*-value less than 0.25. After determining the variables that belonged in our preliminary main effects model, we assessed whether age could be kept in the model as a continuous variable. We found that age in years was not linear and, thus, could not remain as a continuous variable in the model. Using the average age of our sample population as the cut-off point, we dichotomized age into less than 33 years old and greater than 33 years old.

Our final multinomial logistic regression model included covariates for age, gender, education, CVID diagnosis, infection prior to diagnosis, acute illness in the past 12 months, limitation, hospital stay in the past 12 months, hepatitis, other chronic diseases (not counting primary immune deficiency), access to care as needed, primary health care provider specialty, and ever having received regular IVIG treatments. To test how well our model fits the observed values in the IDF questionnaire, we ran the Hosmer-Lemeshow goodness of fit test. The Hosmer-Lemeshow goodness of fit test is a measure of lack of fit of our logistic regression model. Thus, rejection of the null hypothesis for the Hosmer-Lemeshow goodness of fit test indicates that the model we have built adequately fits our data.

When comparing patients who had EVG-PH versus patients who had G-PH, the summary goodness-of-fit statistic was 8.89 (degrees of freedom [*df*] = 8, *p*-value = 0.3519). For patients who had P-PH versus patients who had G-PH, the summary goodness-of-fit statistic was 11.70 (*df* = 8, *p*-value = 0.1653). For both of these strata, the *p*-values indicate that we must reject the null hypothesis that the model is not a good fit to our data. Thus, we can conclude that our model is a good overall fit to the data.

## Results

Of the 1526 PID survey respondents, 61.2 % were adult PID patients, 36.7 % were parents of a PID patient, and 2.1 % were both adult PID patients and parents of a PID patient (Table 1). In cases where there was more than one person with PID in a household, it was necessary to direct the survey recipient on how to select a designated respondent for the survey. Thus, the questionnaire specified that if an adult patient had children with PID, the adult patient should answer the survey questions, rather than their affected children. If there were multiple children with PID in the household, and no adult patients with PID, the parent/caregiver was directed to answer the survey questions about the oldest child with PID. The reason for this selection procedure was to provide a sample with the longest diagnosis and treatment experience.

**Table 1:** Sociodemographic characteristics of respondents (*N* = 1526)

Characteristic	Respondent N (%)
Age (years) Mean 33.2 (±20.2)	
0–6	150 (10)
7–12	195 (13)
13–17	135 (9)
18–29	210 (14)
30–44	315 (21)
45–64	435 (29)
≥ 65	60 (4)
Gender	
Male	638 (42)
Female	880 (57.9)
Education	
8th grade or less	90 (6)
Some high school	45 (3)
Completed high school	270 (18)
Some college	464 (31)
Completed college	345 (23)
Graduate degree	285 (19)
Employment	
Employed	806 (54.3)
Unemployed	325 (21.9)
Other	353 (23.8)
Race	
White, Non Hispanic	1408 (93.7)
Others	95 (6.3)
Respondent	
Adult	931 (61.2)
Parent of a child with PID	558 (36.7)
Both	32 (2.1)
Perceived health	
Excellent/very good	444 (30)
Good	459 (31)
Fair/poor/very poor	601 (39)

**Fig. 2:**
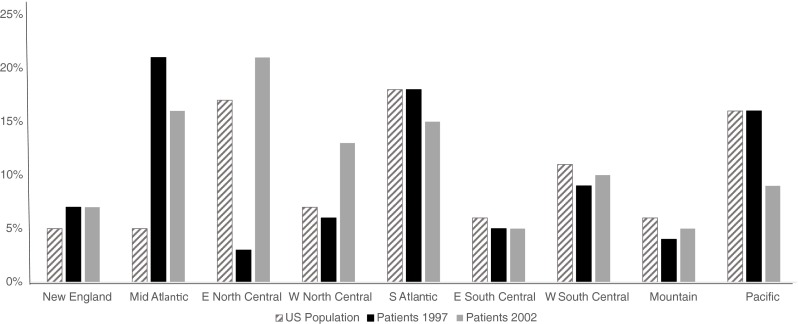
Distribution of 1997 and 2002 National Survey of Patients with PID and overall US population by region

The vast majority of PID patients were White, non-Hispanic (93.7 %). Approximately one-third of the patients were younger than 18 years old and another third were young adults between 18 and 44 years old with mean age of 33 years (SD ±20.2). The geographic distribution of the patient sample closely mirrored the total population of U.S. In comparing the proportion of the U.S. born patients who were born in a particular Census division to the percent of the U.S. population living in that division, the rates were almost identical for New England, Mid-Atlantic, South Atlantic, East and West South Central, and Mountain (Fig. 2).

More than half of the patients were females, which was similar to the general population. The majority of respondents (73 %) had completed college. Fifty four percent of the patients were employed, 21.9 % were unemployed, and the others were homemakers, students, or did not respond with an employment status. Thirty percent of the patients perceived their health status as excellent or very good, 31 % as good, and 39 % as fair, poor or very poor.

A patient’s PH status, which divided the respondents into approximate thirds (excellent/very good, good, and fair/poor), was used to characterize univariate associations between the characteristics of patients with PID. Perceived health was significantly linked to sociodemographic characteristics including age, gender, education level, type of respondent, and employment status (Table 2). When evaluating the sociodemographic characteristics of PID patients with regards to PH 47.5 % of women, but only 29.6 % of men, reported P- PH (*p* < 0.0001). Conversely, a higher percentage of men than women described their health as excellent or very good (39.4 % and 22.4 %, respectively). Older patients were more likely to report P- PH, although this was difficult to separate from reporting by a caretaker as opposed to the patients themselves (*p* < 0.0001). More patients with a college degree had poorer PH when compared to those with less education. Homemakers and students had poorer PH than employed patients and non-homemaker unemployed patients (*p* < 0.0001). Households with a child having PID had better PH when compared to those without any child with PID (39.3 and 22.8 %, respectively, *p* < 0.001). Conversely, households without a child having PID reported poorer PH when compared to those with a child having PID (47.8 and 28.7 %, respectively, *p* < 0.0001).

**Table 2:** Comparison of sociodemographic characteristics of patients with PID by perceived health

	Perceived health					
Demographics	N	Excellent/Very Good %	p	Good * %	Fair/Poor/Very Poor %	p
Age (mean year ± SD)	1479	25.4 (±18.3)	0.0001	31.8 (±20)	39.5 (±19.4)	0.0001
Gender	1497					
Male (%)*		39.4		31.0	29.6	
Female (%)		22.4	0.0001	30.2	47.5	0.0001
Education	1476					
Less than college (%)*		25.5		29.9	44.6	
College & higher (%)		34.8	0.047	31.3	33.9	0.012
Questionnaire respondent	1499					
Adult (%)*		22.9		29.5	47.6	
Parent (%)		44.4	0.0001	31.7	26.9	0.0001
Both (%)		13.3	0.109	43.3	43.3	0.231
Employment	1464					0.0001
Unemployed (%)*		36.5		34.3	29.3	
Employed (%)		36.7	0.99	34.5	28.9	0.900
Other (%)		6.4	0.0001	18.9	74.8	0.0001
No of children with PID in household	1504					
None (%)*		22.8		29.4	47.8	0.0001
At least one (%)		39.3	0.001	32	28.7	0.0001

*Reference

Significance level *p* < 0.05

In addition, PH (in either direction) was significantly associated with a number of other patient characteristics including the number of children with PID in the household, CVID diagnosis, infections prior to diagnosis, reason for initial testing for PID, number of hospitalizations prior to diagnosis with PID, presence of acute illnesses and hospitalization in the past 12 months, limitation of daily physical activity, comorbid conditions, “on demand” access to specialist care, the specialty of the physician caring for the patient (immunologist versus other specialty), and possession of health insurance (Table 3). Having had a visit with immunologist in the past 12 months, the particular setting of physician visits (i.e., doctor’s office, clinic, or hospital), and whether or not the patient has ever received regular IVIG replacement therapy were not significantly associated with PH when considered as individual variables (Table 3).

**Table 3:** Respondent characteristics by perceived health: Represented by distribution of characteristics and statistical significance level

Perceived Health						
		Excellent/very good	p	Good*	Fair/poor/very poor	p
Characteristics	N	%		%	%	
Diagnosis						
CVID	1504		0.003			0.006
	No*	17.1		14.6	15.8	
	Yes	12.4		15.9	24.2	
Family member with PID	1476		0.27			0.91
	No *	22.7		24	31.1	
	Yes	7.3		6.4	8.5	
Infection prior to PID diagnosis	1479		0.002			0.06
	No*	4.3		2.4	2	
	Yes	25.3		28.2	37.8	
Reason for initial testing for PID	1501					
	Family history*	2.8		1.3	1.8	
	Check-up	3.7	0.002	4.9	5.4	0.56
	Recurrent infection	13.5	0.006	14.1	18.1	0.87
	Unusual infections	2	0.48	1.3	2.2	0.54
	Serious infections	3.7	0.001	5.3	6.5	0.75
	Other	3.7	0.025	3.7	6.1	0.55
No of hospitalizations prior to PID diagnosis	1402					
	None*	14.8		13.7	12.6	
	1–3	11	0.81	10.6	9.8	0.97
	4–6	2.4	0.07	3.5	7.3	0.0001
	>6	3.4	0.04	4.9	13	0.0001
Current Health						
Acute illness in past 12 months	1504		0.0001			0.0001
	No*	14		6.2	3.6	
	Yes	15.6		24.3	36.3	
Hospitalization in past 12 months	1500		0.0001			0.001
	No*	24.5		21.2	23.9	
	Yes	5		9.3	16.1	
Limitation of physical activity	1488					
	None*	21		8.3	1.5	
	Slight	7.3	0.0001	14.3	8.4	0.0001
	Moderate	1.1	0.0001	6.5	17.3	0.0001
	Severe	0.3	0.0001	1.2	12.8	0.0001
Comorbidities						
Permanent organ impairment	1504		0.0001			0.0001
	No*	23		2.1	18.9	
	Yes	6.5		9.9	21.1	
Other chronic disease	1467		0.0001			0.0001
	No*	21.1		14.2	9.7	
	Yes	8.9		16.3	29.9	
Cancer/leukemia	1495		0.12			0.004
	No*	28.4		28.8	35.3	
	Yes	1.1		1.9	4.5	
Hepatitis	1483		0.05			0.006
	No	28.7		28.9	35.3	
	Yes	0.9		1.8	4.3	
Neurological disease	1469		0.0001			0.0001
	No	27		25.1	25.9	
	Yes	2.9		5.7	13.5	
Health care access						
Physician specialty	1504		0.24			0.06
	Other	18.5		18	25.8	
	Immunology	11		12.6	14.2	
Setting of primary care visit	1504		0.77			0.52
	Private office*	19.2		20.1	27.1	
	Others	10.3		10.4	12.8	
Access to specialist care as needed	1490		0.0001			0.002
	No*	1.1		3.1	6.8	
	Yes	28.5		27.2	33.3	
Visit with immunologist in past 12 months	1495		0.45			0.80
	No*	9		8.6	11.6	
	Yes	20.6		21.8	28.4	
Insurance status	1487		0.0001			0.002
	Private*	26.2		23.5	21.9	
	Non-private	3.5		6.8	17.1	
	None	0.07		0.3	0.7	
Treatment						
Regular IVIG treatment	1500		0.97			0.11
	No*	6.5		6.8	7.3	
	Yes	23		23.8	32.6	

*Reference

Significance level *p* < 0.05

Since our outcome variable is trinomial (i.e., three levels of PH), we used a multivariate logistic regression analysis approach to determine if there were significant drivers of PH in light of all of the potentially mitigating and confounding variables. These were calculated as odds ratios (OR) together with their 95 % confidence intervals (CI). Using backwards elimination, all covariates with a *p* value of less than 0.25 in the univariate analysis were included in the preliminary multivariate model, and the model was constructed by eliminating the variables, one at a time, based on lack of significance in the presence of other covariates. This method of elimination continued until all variables in the remaining model with either significant or deemed clinically significant. Thus, the final multivariate model included age, gender, education status, having a diagnosis of CVID, infections prior to diagnosis with PID, acute illness and hospitalization in the past 12 months, limitation of physical activity, hepatitis, chronic diseases, “on demand” access to specialist care, the specialty of the physician caring for the patient and having ever received regular IVIG treatment (Table 4). When all of these factors that might impact PH were taken into consideration and adjusted ORs are calculated, significant associations between PH and gender, respondent type, employment status, CVID diagnosis, infections and number of hospitalizations prior to PID diagnosis, reason for initial testing for PID, all comorbidities except chronic diseases, and insurance status were no longer observed.

**Table 4:** Results of multivariate logistic regression: Adjusted ORs relating variables to excellent/very good PH and fair/poor/very poor versus good PH in patients with PID

	*N* = 1353	Excellent/Very Good PH Ref: Good PH		Fair/Poor/Very Poor PH Ref: Good PH	
		OR (95 % CI)	p	OR 95%CI	p
Age (year)	<33*	1.0		1.0	
	≥33	0.68(0.48–0.98)	0.037	1.11(0.78–1.57)	0.562
Gender					
	Male*	1.0		1.0	
	Female	1.02(0.73–1.43)	0.896	0.87(0.62–1.24)	0.447
Education					
	Less than college*	1.0		1.0	
	College & higher	0.65(0.46–0.90)	0.009	1.54(1.12–2.13)	0.008
CVID					
	No *	1.0		1.0	
	Yes	0.72(0.51–1.02)	0.066	1.35(0.96–1.90)	0.086
Infections prior to PID diagnosis					
	No*	1.0		1.0	
	Yes	0.62(0.36–1.07)	0.086	1.66(0.87–3.17)	0.125
Acute illness in past 12 months					
	No*	1.0		1.0	
	Yes	0.45(0.32–0.64)	0.0001	1.20(1.24–3.21)	0.005
Limitation of physical activity					
	None*	1.0		1.0	
	Slight	0.28(0.20–0.40)	0.0001	2.92(1.66–5.14)	
	Moderate	0.13(0.07–0.23)	0.0001	14.16(7.92–25.31)	0.0001
	Severe	0.14(0.04–0.50)	0.003	51.37(24.11–109.41)	0.0001
Hospitalization in past 12 months					
	No*	1.0		1.0	
	Yes	0.53(0.36–0.77)	0.001	1.48(1.05–2.07)	0.025
Hepatitis					
	No*	1.0		1.0	
	Yes	0.51(0.23–1.17)	0.114	1.74(0.93–3.25)	0.081
Other chronic disease					
	No*	1.0		1.0	
	Yes	0.56(0.40–0.78)	0.001	1.70(1.21–2.36)	0.002
Access to specialist care as needed					
	No*	1.0		1.0	
	Yes	2.54(1.27–5.06)	0.008	0.79(0.49–1.27)	0.325
Physician Specialty					
	Others*	1.0		1.0	
	Immunology	0.96(0.69–1.34)	0.813	0.68(0.49–0.94)	0.020
Regular IVIG treatment					
	No*	1.0		1.0	
	Yes	1.61(1.07–2.42)	0.023	1.01(0.67–1.53)	0.951

*Reference

Significance level *p* < 0.05

Some covariates, however, did survive multivariate analyses and thus remain as potentially independent drivers of PH. Age was associated with PH, but only when comparing EVG-PH to G-PH. Respondents 33 years of age and older had lower odds of having EVG- PH (OR 0.68, 95 %CI 0.48–0.98; *p* = 0.037 as opposed to G-PH. Of note, 95.8 % of patients 33 years of age and older were adult patients (data not shown).

Perceived health and education level were significantly associated. PID patients with a college degree were more likely to have P-PH as opposed to G-PH (OR 1.54, 95 %CI 1.12–2.13; *p* = 0.008) and less likely to report EVG-PH (OR 0.65, 95 %CI 0.46–0.90; *p* = 0.009).

The occurrence of acute illness and hospitalization within the last 1 year were significantly associated with P-PH. Patients who were acutely ill and hospitalized in the past 12 months were more likely to have poor as opposed to G-PH (OR 1.20, 95 %CI 1.24–3.21; *p* = 0.005 and OR 1.48, 95 %CI 1.05–2.07; *p* = 0.025, respectively), and less likely to have EVG-PH (OR 0.45, 95 %CI 0.32–0.64; *p* < 0.0001, and OR 0.53, 95 %CI 0.36–0.77; *p* < 0.001, respectively). A strong association was observed between PH and the limitation of physical activity as patients with restrictions had higher odds of reporting P-PH, and lower odds of describing EVG-PH. More strikingly, patients had worse PH if they had more limitation of physical activity. Patients with severe limitation had 51.37 times higher odds of having P-PH (OR 51.37, 95 %CI 24.11–109.41; *p* < 0.001) as opposed to moderate limitation (OR 14.16, 95 %CI 7.92–25.31; *p* < 0.001). The degree of limitation was also relevant as those with slight limitation as opposed to ones without limitation had worse PH (OR 2.92, 95 %CI 1.66–5.14; *p* < 0.0001).

Chronic disease also influenced health perception as patients with chronic disease such as bronchitis, malabsorption, and recurrent infections were more likely to have P-PH (OR 1.70, 95 %CI 1.21–2.36; *p* = 0.002) as opposed to EVG-PH (OR 0.56, 95 %CI 0.40–0.78; *p* = 0.001). Patients with “on demand” access to specialist care had 2.54 higher odds of reporting EVG-PH as opposed to G-PH (OR 2.54, 95 %CI 1.27–5.06; *p* = 0.008). An association between PH and “on demand “access to specialty care, however, was not observed when comparing P-PH to G-PH. Patients who were most often seen by an immunologist for care, however, (when compared to other specialties) were significantly less likely to have P-PH (OR 0.68, 95 %CI 0.49–0.94; *p* = 0.020). Finally, patients who were receiving regular IVIG replacement therapy were more likely to have EVG-PH as opposed to G-PH (OR 1.61, 95 %CI 1.07–2.42; *p* = 0.023).

## Discussion

We used the results from the National Survey of Patients with Primary Immune Deficiency Diseases in America, conducted by the IDF, in an attempt to gain a better understanding of the drivers of PH among patients with PID. Our survey sample was nationally distributed, and based on the single, largest database of persons with PID in the world. Additionally, the sampling frame has always been unique; patients who either have been added to the database since the last patient survey, or those who have never participated in an IDF National Patient Survey before. This provided the ability to track down how the patient population known to IDF, may change over time by administering periodic surveys.

Patients’ own evaluation of their health is a very simple health measure with important links to patient satisfaction and quality of life [28]. As in most other studies, PH was based on the reply to the global question “How would you describe your current health status?” This has been described as a valid, single measure of health status as it is associated with both disease and subjective self-assessment [29,30]. Perceived health has been demonstrated as predictive of objective health measures including disease burden, health care service utilization, and mortality [3,12,31–33]. A large-scale study of PH in PID employing multivariate analyses has to our knowledge not been performed and thus presents the possibility to truly appreciate what affects patient perceptions of health in this rare disease population.

Our results highlight a number of factors as being significant drivers of PH in PID patients including education level, age, acute and chronic disease, hospitalizations, limitation in physical activity, “on demand” access to specialist care, the specialty of the physician caring for the patient, and regular IVIG replacement therapy.

Several studies demonstrate significant associations between sociodemographic factors and PH, including age, gender and socioeconomic status [5,34,35]. The age distribution of the PID patient population, and the mean age of PID patients studied confirm that PID is not simply pediatric condition. In terms of age distribution, the respondents to the 2002 IDF survey are slightly different from other existing databases and registries [36]. The registries aim to cover all the cases occurring in the country, but age distribution in registries depends on the nature of the contributing centers with most of the cases diagnosed in children (e.g., the median age at diagnosis in the French registry is 3.3 years) in some registries, whereas others have a majority of adult cases (e.g., the median age in the Australian registry is 31 years) [37]. Comparing IDF database to USIDNET, another U.S. database, the current age of the patients in the USIDNET registry is 30 years. Based on the IDF data, the average age at which patients were diagnosed with PID was the same as to what the current age of the patient was in USIDNET, which was 30 years (personal communication). So the average age of PID patients in both U.S. databases are similar to each other.

In our study, age was a significant determinant of PH among PID patients, but only when comparing EVG-PH to G-PH. Our finding suggests that older patients are less likely to have EVG-PH compared to G-PH. This may be due to the fact that in general elderly are more likely to have health problems [33], therefore likely not perceiving their health as excellent or very good. There are other cross-sectional, population based studies reporting of good PH decreased with age [38,39]. In addition to age, the only other sociodemographic characteristic, which was associated with PH in our study, was education level. Patients with college and higher education were more likely to report P-PH. There are inconsistent findings regarding the link between PH and education status. Our result is in contrast with some published literature [40,41] but consistent with few others [42]. It has been suggested that individuals with higher education might have more health knowledge or contact with health services, making them more accurate predictors of their own health status. Highly educated individuals, however, may have higher expectations about their quality of life and in some research have been linked to being more frequently dissatisfied with their health [43]. In addition, people with higher education levels have been documented as having greater stress levels, which could have a negative impact on PH [24]. In fact, a “dose–response” relation between PH and stress is well documented [44].

Chronic diseases are a particular concern within medicine and have been subject of substantive research as they may last for a patient’s entire lifetime, require multiple physician visits, have acute exacerbations necessitating hospital admissions, and leave sequelae. Our findings in PID patients align with previous reports, as patients with chronic disease were more likely to have P-PH compared to patients who reported G-PH. They were also far less likely to report EVG-PH when compared to patients reporting G-PH. Several reports document negative impact of chronic disease on PH [45,46]. In addition, PH can predict onset of chronic disease [12]. In a longitudinal study of healthy late midlife U.S. adults, respondents with higher PH at baseline were less likely to experience subsequent first-time chronic disease including arthritis, diabetes, stroke, coronary heart disease, and lung disease [47]. The relative impact of different chronic diseases on the level of PH has been investigated and results differ depending on the condition evaluated [23,48]. As an example, chronic diseases including gastrointestinal, neurological, renal and musculoskeletal disorders had more negative impact on PH compared to some other chronic diseases such as asthma and diabetes [49]. As an important limitation and like other data in our study, chronic conditions were self-reported. These self reports also did not address individual chronic diseases comprehensively, which would be important to discern as particular chronic conditions have the potential to contribute substantively to PH.

Hospital admission is a known significant driver of PH and visa-versa and thus the fact that we identified it as an influence upon PH in PID is not a surprise. In a cross-sectional study of 1678 older diabetes patients PH status was an independent predictor of hospitalization in the following year [50]. A longitudinal study in Sweden, identified associations between PH and hospital admission in men, but not women, which were attributed to the age differences and follow-up time [51].

Physical health, as might be expected, has consistently been identified as a major predictor of PH [52,53]. Longitudinal as well as cross-sectional studies have defined a significant link between acute illness and PH independent of the subjects’ chronic illnesses, which are in line with our findings [54,55]. On another note, impact of acute illnesses on PH may not be significant in younger and generally healthy patients [56].

We also found that limitation of physical activity was a very strong predictor of PH in PID. Furthermore, the degree of the limitation had significant impact on PH with increasing activity limitation significantly decreasing the odds of G-PH. This suggests that the largest contribution to PH among PID patients derives from the extent to which patients can do what they need and want to do. This finding is consistent with results of several studies performed in other diseases [57,58]. Along these lines, it has been reported that a patient’s perception of disease related to musculoskeletal system was the best predictor of PH in both men and women [59], and the impact of limitations increases with age [60]. Importantly daily activity is an indicator of independent living [61], and limitation can also predict decline in PH across age groups [62]. Thus, physical activity and perceived limitation likely represent an important focus area to promote improved PH in PID patients and warrant further targeted study.

Our observation of the strong influence of “on demand” access to specialist care upon a PID patient’s PH is also consistent with previous studies in other diseases [63]. When compared to other measures of patient satisfaction such as provider and quality of care received, access to care was found to be more strongly associated with PH [28]. Moreover, although not consistent across the literature [64] patients who visited specialists were more satisfied with their health status [28]. We observed only a weak association between PH and physician specialty with patients seeing an immunologist reporting less P-PH. This suggests at least some importance to PID patients having access to specialized clinical immunologists with regards to their optimal PH.

Many patients with PID are faced with the challenge of life-long therapy with regular IgG replacement intravenously or subcutaneously in order to reduce susceptibility to and severity of infections. Studies demonstrated improved PH and health related quality of life after initiation of IgG replacement therapy, which was attributed to decreased infection frequency [65,66]. Similarly, we found that regular IVIG replacement was significantly associated with EVG-PH. The fact that a specific and indicated therapy links to improved PH is important to be cognizant of in making treatment decisions as PH is a strong indicator of future health [58]. Thus patients for whom IgG replacement therapy is indicated should be provided this therapy.

Despite the underlying immune deficiency, about two thirds of patients in our study reported EVG-PH. A previous study of adults with XLA found that with the exception of their PH, patients had a comparable quality of life to that of the general population [26]. This suggests that although XLA has impact on patients’ daily lives as determined by missed school/work days or hospitalizations, they can be moderately healthy and lead productive lives [67]. On the contrary, in a study of pediatric XLA patients, perceived psychosocial health was poor despite good perceived physical health [27]. That said, P-PH and reduced family activities in children with PID have been previously identified and attributed to frequent, or serious infections and limitations of physical activity [68]. Differences between adults and children with PID may partially be explained by age-related adaptation and development of coping mechanisms, thus presenting potential strategies to explore through additional research to improve PH. Perceived health is also likely to be a feature of the underlying PID as PH has been found to be lower in CVID as opposed to XLA patients [67,69].

Our findings capture PID patients’ own experiences and views of their health in the context of their disease. As such, they indicate that clinical practice should ensure that treatment plans and evaluations focus on the patient rather than the disease. In fact, clinicians could use PH assessment as a tool to uncover patients’ problems, and unmet health needs. The potential benefit of using PH measures in clinical practice is that after problems are identified, further care decisions could be linked to patients’ priorities and preferences [70]. This can positively drive PH, which is again a significant predictor of future health [58].

In a large study of adults positive PH changes were associated with better functional well-being and greater survival odds later in life [71]. Unden et al. suggested that irrespective of physician’s rating of patient’s health during an office visit, those with “poor” PH, perceived lower social and mental well-being, more somatic conditions, and worse coping abilities [10]. Perceived health measures seem to make it practical to look beyond traditional measures of biological functioning to larger issues of functioning and well-being.

Knowledge of a patient’s PH could allow for clinical planning that takes into account all of the patient’s needs. As some drivers of PH, including chronic/acute disease, and limitation of physical activity significantly influenced PH, the establishment of multidisciplinary health care teams to provide better outpatient and home care services might be helpful for PID patients and certainly worthy of further study with regards to PH. We would suggest that better recognizing the factors that drive PH in PID patients can guide improvements in clinical care and help identify patient health needs. For example, given our findings, interventions to increase physical activity may help improve patients’ PH. Similarly; improving health-care access to an immunologist might serve the same purpose. Based upon our findings, we also suggest a distinct potential value to regular preventive screenings to preempt an acute disease, as well as encouragement of regular IgG replacement therapy for patients in whom it is indicated. These should all be considered as possible ways to improve PH in PID patients, which again is a known of predictor of future health [58]. In addition, we would recommend a single question assessing a patient’s PH be incorporated into the routine data collected during a clinic visit to allow predictive healthcare modeling.

To the best of our knowledge, this is the first study analyzing PH in general among patients, and parents of patients with a variety of PID diagnoses based on a national U.S. sample. One of the strengths of our study was that it was based on data derived from a national patient-based survey and data covered several self-reported medical history items. It also captured the respondents’ own views of their health. There are limitations, however, including that we used a cross-sectional survey, which makes it difficult to establish the causal relationship between PH and the factors identified. Because of the cross-sectional nature of the survey, analyses were limited to the PID diagnoses present at the time of the survey and the impact of other domains specific to that time. Accordingly, our discussion of the trajectories does not contain a comparison group. Also, the data upon which our study was based was self-reported and not independently verified. Thus, there is a possibility of information bias. Finally, selection bias, may have selected for respondents that may have been more health conscious, and therefore, more likely to report better PH. Alternatively, non-respondents may have been in poor health. Although random assignment is preferable for unbiased population estimates, and pediatric survey instruments are available, the survey our data was based on did not target children directly to fill out the distributed surveys. Instead parents/care givers were asked to respond on behalf of children. This survey procedure also introduces some selection bias towards older respondents in households with multiple individuals with PID. The selection bias may be minimized utilizing a pediatric survey tool for future studies. In some cases in surveys‚ one might ask the same questions of the parent-proxy and of the child. This essentially means having at least two surveys completed for each minor child, one from the proxy and one from the child. Although it might be interesting to compare the results, determining which answer to use if there are differences between responses for a child is problematic. Additionally, the cost involved to conduct the survey with multiple instruments for a family, both in complexity, potential respondent burden and in true monetary costs would be beyond the scope of the goals for the survey.

Our results, however, define some relevant signal and create rationale for further evaluation of PH in PID. We propose that a longitudinal study is needed to further clarify contribution of various factors to PH of patients with PID.

The strength of measures of general health perceptions lies in their subjectivity and that patient values will help shape the goals and means of medical care [72]. Our results suggest that clinical care of patients with PID should be tailored to the patients’ specific characteristics and needs to achieve better patient outcomes. As patient outcomes research progresses, each step in this direction brings medicine closer to pursuing “what really matters to our patients?” rather than “what is the matter with our patients?”
